# The dating of the fourth volume of Guillaume-Antoine Olivier’s “Entomologie, ou histoire naturelle des insectes”

**DOI:** 10.3897/zookeys.734.22901

**Published:** 2018-02-05

**Authors:** Yves Bousquet

**Affiliations:** 1 Gatineau, Quebec, Canada

**Keywords:** Coleoptera, beetles, date of publication, literature

## Abstract

Despite the title page is dated 1795, the fourth volume of Olivier’s *Entomologie, ou histoire naturelle des insectes* was issued in two parts, one probably in 1795 and the second in 1800. All new taxa made available in this work have previously been dated 1795 in the literature. A list of new species described in 1795 and a list of those that have to be dated 1800 are appended. The genus *Necrobia* should be credited to Latreille, 1797, not Olivier, 1795.

Born in the commune Les Arcs (also known as Les Arcs-sur-Argens), a small village near Toulon in the Var department, southeastern France, on 19 January 1756, Guillaume-Antoine Olivier (Fig. 1) was a French physician and naturalist. He studied medicine at Montpellier and at 17 years old practiced medicine in his native town but soon found his job uninteresting and poorly paid. In 1783, he moved to Paris and worked for Louis Bénigne François Bertier de Sauvigny (b. 1737; d. 1789), the intendant of Paris, and conducted a statistical survey on the generality of Isle de France. Later he was hired by Jean-Baptiste Gigot d’Orcy (b. 1737; d. 1793), the wealthy finance receiver general, to write a natural history of the insects and this is the reason behind Olivier’s connection with the *Entomologie ou histoire naturelle des insectes*. For this project, Olivier travelled to Britain and the Netherlands to describe the insects and have them illustrated. At about the same time, Olivier was approached to contribute to the natural history of the insects for Charles-Joseph Panckoucke’s (b. 1736; d. 1798) *Encyclopédie méthodique*, one of the major scientific publication achievements of all time (Evenhuis 2003). In October 1792, Olivier and his friend Jean Guillaume Bruguière (b. 1749/1750; d. 1798) were chosen by the French government to take part in a scientific and diplomatic mission to the Ottoman Empire, Egypt, and Persia. The two sailed from Marseille in April 1793 and for the next six years visited many places in the Middle East where they had the opportunity to collect natural history specimens. Olivier returned to France in December 1798 while Bruguière died in Ancôme on the journey back. Upon his return, Olivier became a member of the prestigious *Académie des Sciences* in 1800 and worked mainly at writing his two major entomological works and the account of his trip, which was published in three volumes of text in quarto and one volume of plates in 1801, 1804, and 1807. In 1811, he was appointed professor of zoology at *L’École nationale vétérinaire d’Alfort* but soon suffered from anaemia (wasting disease). In 1814, he went to his native town to rest and on his way back stopped at Lyon where he was found dead, from an aortic aneurism, in his bed on October 1. He was 58 years old. Olivier was a close friend to Johan Christian Fabricius and a patron to Pierre André Latreille particularly during the French Revolution. This account of Olivier’s life is derived from Cuvier (1818) and Walckenaer (1830).

**Figure 1.**
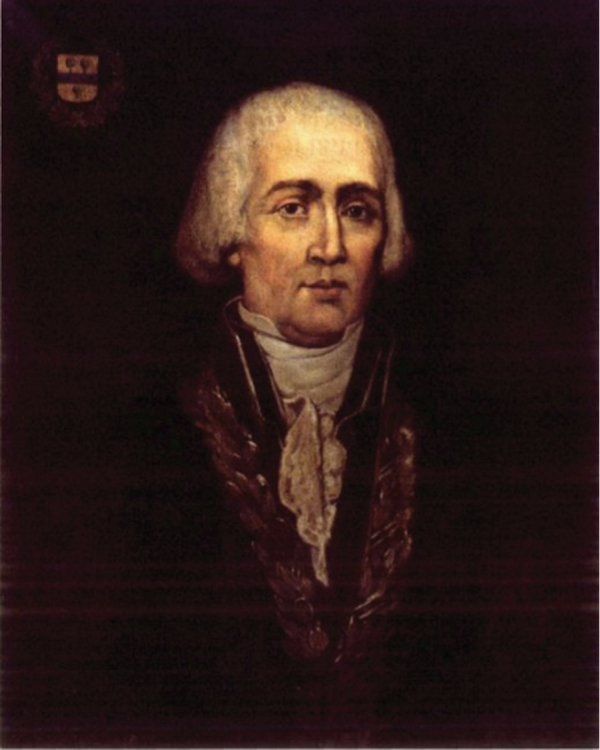
A photographic reproduction of an oil portrait of Guillaume-Antoine Olivier [source: Bernard (1997: fig. 1)].

One of the two major publications of Olivier is his *Entomologie ou histoire naturelle des insectes*. As the title suggests, Olivier apparently intended to treat all insect orders, but in the end only the Coleoptera were dealt with. Six volumes of text and two of plates were published between 1789 and 1808. Each genus in the first four volumes was given a number and separately paginated. The entire work consisted of 3,162 pages and 363 plates (either black and white or colored) issued in 30 livraisons (Anonymous 1808).

The fourth volume treated 18 genera: No 66, Prione / *Prionus* (41 pp.); No 67, Capricorne / *Cerambix* [*Cerambyx*] (132 pp.); No 68, Saperde / *Saperda* (41 pp.); No 69, Stencore / *Stenocorus* (30 pp.); No 70, Callidie / *Callidium* (72 pp.); No 71, Spondyle / *Spondylis* (4 pp.); No 72, Calope / *Calopus* (4 pp.); No 73, Lepture / *Leptura* (34 pp.); No 74, Nécydale / *Necydalis* (10 pp.); No 74*bis*, Cucuje / *Cucujus* (10 pp.); No 75, Donacie / *Donacia* (12 pp.); No 75*bis*, Lupère / *Luperus* (4 pp.); No 76, Clairon / *Clerus* (18 pp.); No 76*bis*, Nécrobie / *Necrobia* (6 pp.); No 77, Bostriche / *Bostrichus* (18 pp.); No 78, Scolyte / *Scolytus* (14 pp.); No 79, Bruche / *Bruchus* (24 pp.); No 80, Macrocéphale / *Macrocephalus* (16 pp.). Its title page is dated 1795 (Fig. 2) and all publications seen citing the volume have dated it as 1795. However, *livraison* 23 of the *Entomologie ou histoire naturelle des insectes* by the citoyen Olivier was announced on 14 Fructidor an 8 (= 31 August 1800) in the *Gazette Nationale ou Le Moniteur Universel* and in the Fructidor an VIII (= 18 August–22 September 1800) issue of the *Journal Général de la Littérature de France*, both journals recording new books published in France. The citation mentioned that the continuation of Olivier’s work was postponed because of the six-year voyage of the author in the Orient, and that the present livraison contains about three-quarters of the fourth volume, including the explanatory text of 56 plates. There is other evidence that part of Olivier’s fourth volume of his *Entomologie* was issued after 1795. The work contains five explicit references to Fabricius’ *Supplementum entomologiae systematicae* which was published in 1798: “Lamia bicincta. Fab. suppl. Ent. Syst. pag. 145” under Capricorne continu (No 67, p. 123), “Lamia marmorata. Fab. Suppl. Ent. Syst. pag. 144. n^o^.1” under Capricorne bigarré (No 67, p. 124), “Cucujus rufus. Fab. Suppl. Ent. Syst. emend. pag. 123” under Cucuje fauve (No 74*bis*, p. 5), “Lema flavipes. Fab. Suppl. Ent. Syst. pag. 93. n^o^. 21” under Lupère flavipède (No 75*bis*, p. 4), and “Anthribus *niveirostris* rostro latissimo plano elytrorumque apicibus anoque albis. Fab. Ent. Syst. Suppl. pag. 160” under Macrocéphale nivéirostre (No 80, p. 8). In addition, on page 121 (No 67, footnote), Olivier mentioned “Ce genre ayant été imprimé pendant mon voyage dans les contrées orientales, on a omis quelques descriptions que je m’empresse de donner ici” [This genus was printed during my voyage to the oriental region and some descriptions were omitted which I hasten to present here]. As mentioned previously, Olivier returned from his trip in December 1798. Finally, Illiger (1800: ix) mentioned in the *Vorrede*, dated 15 April 1800, of the first volume of his German translation of Olivier’s work “Entomologie” that the fourth volume of the series was not yet published [*Der vierte noch nicht erschienene Band wird wahrscheinlich der Werk schliessen*].

**Figure 2.**
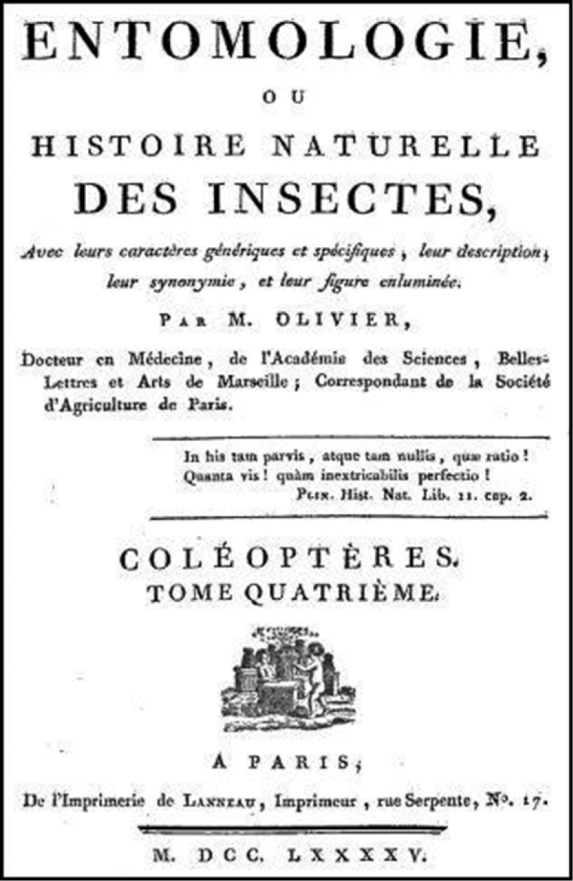
Title page of Olivier’s tome 4 of the *Entomologie, ou histoire naturelle des insectes*.

One problematic question remains. What exactly is the content of *livraison* 23 since the recording journals cited above simply mentioned that it included about three-quarters of the fourth volume? Bousquet (2016: 393) surmised that it could comprise the text from page 81 of the Capricorne (No 67). In fact there is a clue in the text suggesting that this could be the case. All capricorne species described up to page 80 have the Latin generic name incorrectly spelled *Cerambix*, while those on the following pages have the name correctly spelled *Cerambyx*. This is circumstantial evidence that a break occurred in the printing of the text. So, as far as I am concerned all new species described from page 81 (No 67) onwards should be dated 1800. A list of them is given in Appendix 2.

The genus *Necrobia* has been attributed to “Olivier 1795” from this work by almost all authors seen. The name is so entered in the *Official List of Generic Names in Zoology* following Opinion 604 (ICZN 1961). However, this is incorrect since the name appeared in *livraison* 23 of Olivier’s *Entomologie* which, as previously mentioned, was published in 1800. Olivier (1800: 1, No 76*bis*) wrote under *Necrobia* “Le cit. Latreille est le premier qui ait senti que ces insectes [*Clerus*] devoient être séparés des uns et des autres, et former un genre particulier, auquel il a donné le nom de Nécrobie...” [Latreille is the first that saw the necessity to separate these insects (referring to those of the genus *Clerus*) and formed a peculiar genus to which he gave the name Necrobie (i.e., *Necrobia*)^1^]. Latreille (1797: 35) indeed first proposed the name *Necrobia* and made it available. This was recognized by Sherborn (1902: 650) who correctly credited the genus from Latreille’s *Précis des caractères génériques des insectes* while Neave (1940: 276) wrote beside *Necrobia* “Olivier 1795 [?], Entomologie 4, no. 76 (*bis*); Latreille 1796, Préc. Car. Ins., 35.” Although Latreille described the genus, he did not include any species under it. The nominal species first subsequently and expressly included in the genus *Necrobia* are the three cited by Olivier (1800), namely *N. violacea*, *N. rufipes*, and *N. ruficollis*. In Opinion 604 (ICZN 1961), *Dermestes violaceus* Linnaeus, 1758 was validated as the type species of the genus.

There are 72 plates associated with the genera treated in volume 4 of Olivier’s *Entomologie*: 13 for *Prionus* (No 66), 23 for *Cerambix / Cerambyx* (No 67), 4 for *Saperda* (No 68), 3 for *Stenocorus* (No 69), 8 for *Callidium* (No 70), 1 for *Spondylis* and *Calopus* (Nos 71 and 72); 4 for *Leptura* (No 73); 1 for *Necydalis* (No 74); 1 for *Cucujus* (No 74*bis*); 1 for *Donacia* and 1 for *Donacia* and *Luperus* (Nos 75 and 75*bis*); 1 for *Clerus* and 1 for *Clerus* and *Necrobia* (Nos 76 and 76*bis*); 3 for *Bostrichus* (No 77); 2 for *Scolytus* (No 78); 3 for *Bruchus* (No 79); 2 for *Macrocephalus* (no 80). There are no scientific names on the plates^2^, except for the respective genus at the top. At the recommendation of the editor, these plates were usually placed in the eighth volume (the second of the plates) of the series. The title page is dated 1808 but it is obvious that most, if not all, of the plates were issued prior to this date. For example, Fabricius (1792) cited the following plates from Olivier’s volume 4: 1–6, 12 (*Prionus*), 1–12, 14–19 (*Cerambyx*), 1 and 2 (*Saperda*), 2 (*Stenocorus*), 1–7 (*Callidium*), 1 and 2 (*Leptura*).

The question remaining is when the first part of Olivier’s volume 4 (i.e., Nos 66 and 67 up to page 80) was actually published. I have been unable to find the *livraison* in which this part was published nor did I find a date of publication in a recording journal for livraison 22 of the work, which could deal with the first part. *Livraison* 21 was noticed in 1796 in the third volume of the second year of the *Magasin Encyclopédique ou Journal des Sciences, des Lettres et des Arts* (p. 558) but no indication was provided as to its content. What is puzzling is that the first author I found giving reference to any of the species included in the entire fourth volume is Latreille (1804), more than eight years after the alleged publication of the first part in 1795. Even Fabricius (1801), who intended to treat all Coleoptera known at the time, did not include any of the new species described in Olivier’s entire fourth volume of his *Entomologie*. Since the title page is dated 1795 (Fig. 2), the date of 31 December 1795 should be adopted as the correct date of publication of the first part (ICZN 1999, Article 21.3.2) until additional evidence is found. A list of the new species described in the first part is included in Appendix 1.

## Footnotes

1 It is ironic that Latreille proposed the generic name for the insect [*Necrobia ruficollis* (Fabricius)] that saved his life a few years prior when he was jailed as a non-jurist priest waiting for deportation (see Peyerimhoff 1932: 66–67, for the story). Latreille’s friends, who paid for his monument, had the figure of the beetle engraved along with these words “*Necrobia ruficollis*, Latreillii salus” (Lemaout 1842: 322).

2 The copy on Biodiversity Heritage Library has handwritten specific names added subsequently.

## Appendix 1

List of new species described in Olivier’s first part of volume 4 of his *Entomologie* (Nos 66 and 67 up to p. 80), dated 1795, along with the original localities mentioned. Note. *Cerambix denticornis* [67: 60; pl. 5, fig. 33] is a replacement name for *Lamia spinicornis* Fabricius, 1781 and *Cerambix sulcatus* [67: 28; pl. 16, fig. 113] is a replacement name for *Cerambyx festivus* Fabricius, 1775.

*Prionus accentifer* [66: 8; pl. 4, fig. 16] [[Locality not indicated]]

*Prionus angulatus* [66: 31; pl. 1, fig. 2] [Locality not indicated]

*Prionus ater* [66: 11; pl. 7, fig. 24] Cayenne

*Prionus castaneus* [66: 23; pl. 8, fig. 28, 29] [Locality not indicated]

*Prionus cinereus* [66: 35; pl. 13, fig. 55] Surinam

*Prionus corticinus* [66: 21; pl. 9, fig. 34] Cayenne

*Prionus crenatus* [66: 27; pl. 12, fig. 45] Cayenne

*Prionus exsertus* [66: 17; pl. 8, fig. 31] Saint-Domingue

*Prionus maculatus* [66: 27; pl. 4, fig. 14] Sénégal

*Prionus obscurus* [66: 26; pl. 1, fig. 7] Provence

*Prionus octangularis* [66: 33; pl. 6, fig. 19 + pl. 13, fig. 54] [Locality not indicated]

*Prionus orientalis* [66: 28; pl. 13, fig. 51] Ceylan

*Prionus quadrilineatus* [66: 40; pl. 3, fig. 11] [Locality not indicated]

*Prionus scutellaris* [66: 14; pl. 2, fig. 9] Cayenne

*Prionus senegalensis* [66: 22; pl. 7, fig. 25] Sénégal

*Prionus sericeus* [66: 16; pl. 8, fig. 26] Cayenne

*Prionus serraticornis* [66: 14; pl. 9, fig. 33] [Locality not indicated]

*Prionus speciosus* [66: 31; pl. 4, fig. 13] [Locality not indicated]

*Prionus sulcatus* [66: 39; pl.8, fig. 27] Cayenne

*Prionus tuberculatus* [66: 20; pl. 6, fig. 22] Amérique méridionale

*Prionus undatus* [66: 32; pl. 13, fig. 53] Surinam

*Prionus vittatus* [66: 39; pl. 6, fig. 20] Indes orientales

*Cerambix analis* [67: 37; pl. 19, fig. 144] [Locality not indicated]

*Cerambix angolator* [67: 71; pl. 22, fig. 170] Angole

*Cerambix bicinctus* [67: 46; pl. 21, fig. 166] [Locality not indicated]

*Cerambix bilineatus* [67: 17; pl. 21, fig. 161] [Locality not indicated]

*Cerambix crassicornis* [67: 51; pl. 20, fig. 150] [Locality not indicated]

*Cerambix emarginatus* [67: 48; pl. 22, fig. 82] Brésil

*Cerambix fuliginosus* [67: 14; pl. 10, fig. 64] [Locality not indicated]

*Cerambix globosus* [67: 27; pl. 12, fig. 81] Batavia

*Cerambix hirtipes* [67: 36; pl. 20, fig. 157] Cap de Bonne-Espérance

*Cerambix humeralis* [67: 38; pl. 19, fig. 141] [Locality not indicated]

*Cerambix maculatus* [67: 68; pl. 7, fig. 49 + pl. 22, fig. 174] Indes orientales

*Cerambix maxillosus* [67: 52; pl. 20, fig. 147] [Locality not indicated]

*Cerambix nigripes* [67: 52; pl. 20, fig. 149] [Locality not indicated]

*Cerambix papulosus* [67: 72; pl. 20, fig. 156] Indes orientales

*Cerambix rugosus* [67: 12; pl. 21, fig. 159] Cayenne

*Cerambix scapularis* [67: 17; pl. 21, fig. 162] [Locality not indicated]

*Cerambix scutellaris* [67: 16; pl. 21, fig. 160] [Locality not indicated]

*Cerambix subocellatus* [67: 69; pl. 2, fig. 12] [Locality not indicated]

*Cerambix unidentatus* [67: 20; pl. 19, fig. 145] [Locality not indicated]

*Cerambix verrucosus* [67: 63; pl. 20, fig. 148] [Locality not indicated]

*Cerambix virescens* [67: 77; pl. 2, fig. 8] [Locality not indicated]

## Appendix 2

List of new species described in Olivier’s second part of volume 4 of his *Entomologie* (from No 67 page 81 to the end), issued in 1800, along with the original localities mentioned.

*Cerambyx aestuans* [67: 123; pl. 23, fig. 176] Sénégal

*Cerambyx armatus* [67: 121; pl. 19, fig. 14] Surinam

*Cerambyx bifasciatus* [67: 94; pl. 14, fig. 98] Afrique équinoxiale

*Cerambyx caelatus* [67: 99; pl. 11, fig. 79 + pl. 12, fig. 79] Indes orientales

*Cerambyx continuus* [67: 123; pl. 23, fig. 177] Afrique

*Cerambyx crocatus* [67: 92, pl. 12, fig. 80] Madagascar

*Cerambyx decorus* [67: 128; pl. 5, fig. 38] Sénégal

*Cerambyx dentifer* [67: 132; pl. 23, fig. 185] Afrique

*Cerambyx didymus* [67: 125; pl. 23, fig. 179] Amérique méridionale

*Cerambyx formosus* [67: 86, pl. 20, fig. 153] [Locality not indicated]

*Cerambyx gallo-provincialis* [67: 125, pl. 3, fig. 17] Provence

*Cerambyx hemipterus* [67: 127; pl. 23, fig. 181] Java

*Cerambyx lateralis* [67: 129; pl. 5, fig. 36] [Locality not indicated]

*Cerambyx macularis* [67: 98; pl. 20, fig. 154] Surinam

*Cerambyx obsoletus* [67: 130; pl. 13, fig. 90] Caroline, Pensylvanie

*Cerambyx ornatus* [67: 88; pl. 4, fig. 24 + pl. 1, fig. 6] Afrique

*Cerambyx pectoralis* [67: 122; pl. 23, fig. 175] Sénégal

*Cerambyx plumosus* [67: 98; pl. 20, fig. 152] Indes orientales

*Cerambyx sanguinolentus* [67: 93; pl. 20, fig. 155] [Locality not indicated]

*Cerambyx sordidus* [67: 124; pl. 1, fig. 5] Sénégal

*Cerambyx spinipes* [67: 103; pl. 10, fig. 66] isle de Bourbon

*Cerambyx stigma* [67: 126; pl. 23, fig. 180] Amérique méridionale

*Cerambyx umbraticus* [67: 129; pl. 11, fig. 75] Cayenne

*Cerambyx villicus* [67: 102; pl. 10, fig. 72] isle de Bourbon

*Saperda annularis* [68: 11; pl. 4, fig. 36] Espagne

*Saperda bicolor* [68: 32; pl. 3, fig. 25] Amérique septentrionale, en Géorgie

*Saperda bicornis* [68: 27; pl. 4, fig. 46] [Locality not indicated]

*Saperda bimaculata* [68: 21; pl. 4, fig. 43] Département du Var

*Saperda cinerea* [68: 28; pl. 3, fig. 35] Amérique septentrionale

*Saperda cornuta* [68: 26; pl. 4, fig. 45] Surinam

*Saperda elegans* [68: 15; pl. 4, fig. 40] [Locality not indicated]

*Saperda elongata* [68: 19; pl. 3, fig. 34] Chine

*Saperda fasciculata* [68: 14; pl. 1, fig. 3] Amérique méridionale

*Saperda filiformis* [68: 28; pl. 4, fig. 47] Sénégal

*Saperda hirticollis* [68: 11; pl. 4, fig. 37] [Locality not indicated]

*Saperda hirtipes* [68: 14; pl. 1, fig. 8] Amérique méridionale, Cayenne, Surinam

*Saperda lunaris* [68: 7; pl. 2, fig. 21] Indes orientales

*Saperda maculata* [68: 32 + 68: 39; pl. 3, fig. 33] Amérique septentrionale, dans la Géorgie

*Saperda mucronata* [68: 30; pl. 1, fig. 10] [Locality not indicated]

*Saperda pallipes* [68: 31; pl. 4, fig. 49] Surinam

*Saperda plumbea* [68: 21; pl. 4, fig. 42] Amérique septentrionale

*Saperda rufipes* [68: 25; pl. 2, fig. 14] Département du Var

*Saperda thoracica* [68: 18; pl. 2, fig. 19] [Locality not indicated]

*Saperda tridentata* [68: 30; pl. 3, fig. 48] Canada

*Stenocorus bicolor* [69: 16; pl. 1, fig. 4] [Locality not indicated]

*Stenocorus humeralis* [69: 22; pl. 2, fig. 18] Allemagne et en Hongrie

*Stenocorus laevis* [69: 21; pl. 3, fig. 25] France

*Stenocorus lineatus* [69: 13; pl. 3, fig. 22] Amérique

*Stenocorus niger* [69: 19; pl. 3, fig. 24] Mont-Pila

*Stenocorus scrutator* [69: 10; pl. 3, fig. 21] Autriche

*Stenocorus sericeus* [69: 20; pl. 1, fig. 8] France

*Stenocorus suturalis* [69: 29; pl. 4, fig. 29] Indes orientales

*Stenocorus testaceus* [69: 27; pl. 2, fig. 20] Cap de Bonne-Espérance et dans la Géorgie

*Callidium abdominale* [70: 70; pl. 8, fig. 103] midi de la France

*Callidium araneiforme* [70: 61; pl. 7, fig. 90] Saint-Domingue

*Callidium arvicola* [70: 64; pl. 8, fig. 93] midi de la France

*Callidium campestre* [70: 65; pl. 8, fig. 95] Amérique septentrionale

*Callidium cinereum* [70: 69; pl. 8, fig. 102] Saint-Domingue

*Callidium decorum* [70: 63; pl. 8, fig. 92] Newyork

*Callidium irroratum* [70: 70; pl. 8, fig. 104] Saint-Domingue

*Callidium lucidum* [70: 59; pl. 7, fig. 86] Saint-Domingue

*Callidium notatum* [70: 61; pl. 7, fig. 89] New-York

*Callidium palmatum* [70: 29; pl. 7, fig. 82] Amérique méridionale

*Callidium pini* [70: 71; pl. 8, fig. 105] New-York

*Callidium pulverulentum* [70: 69; pl. 8, fig. 101] Amérique septentrionale

*Callidium rhombifer* [70: 46; pl. 4, fig. 51] Géorgie

*Callidium rufum* [70: 28; pl. 7, fig. 81] [Locality not indicated]

*Callidium ruricola* [70: 65; pl. 8, fig. 96] Saint-Domingue

*Callidium spinicorne* [70: 68; pl 8, fig. 100] Saint-Domingue

*Callidium suturale* [70: 62; pl. 7, fig. 91] Saint-Domingue

*Callidium unicolor* [70: 58; pl. 7, fig. 84] côtes de Barbarie; Asie mineure, dans la Mésopotamie

*Callidium verrucosum* [70: 67; pl. 8, fig. 98] New-York

*Callidium villicum* [70: 64; pl. 8, fig. 94] Amérique septentrionale

*Leptura acuminata* [73: 20; pl. 3, fig. 35] Amérique septentrionale

*Leptura arcuata* [73: 32; pl. 4, fig. 47] Amérique septentrionale

*Leptura canadensis* [73: 8; pl. 3, fig. 27] Canada

*Leptura circumdata* [73: 32; pl. 4, fig. 48] Amérique septentrionale

*Leptura cordifera* [73: 25; pl. 4, fig. 41] Amérique septentrionale

*Leptura cruciata* [73: 7; pl. 1, fig. 5] environs de Paris

*Leptura decem-punctata* [73: 26; pl. 4, fig. 42] Hongrie, aux environs de Paris

*Leptura lateralis* [73: 22; pl. 3, fig. 37] Amérique septentrionale

*Leptura limbata* [73: 31; pl. 2, fig. 20] Europe

*Leptura notata* [73: 11; pl. 1, fig. 11] Europe

*Leptura vagans* [73: 31; pl. 4, fig. 46] Amérique septentrionale

*Leptura velutina* [73: 18; pl. 3, fig. 32] Amérique septentrionale

*Leptura vittata* [73: 30; pl. 4, fig. 45] Canada

*Leptura zebra* [73: 19; pl. 3, fig. 33] Amérique septentrionale

*Necydalis abdominalis* [74: 8; pl. 1, fig. 5] Cayenne

*Necydalis analis* [74: 7; pl. 1, fig. 4] [Locality not indicated]

*Necydalis fasciata* [74: 10; pl. 1, fig. 9] Amérique méridionale

*Necydalis nigricornis* [74: 10; pl. 1, fig. 8] Surinam

*Necydalis sanguinicollis* [74: 9; pl. 1, fig. 7] Amérique septentrionale

*Cucujus americanus* [74*bis*: 7; pl. 7, fig. a.b.] Cayenne

*Cucujus ater* [74*bis*: 9; pl. 1, fig. 10.a.b.] Europe

*Donacia caerulea* [75: 10; pl. 2, fig. 10] Caroline

*Donacia palmata* [75: 8; pl. 1, fig. 7] Amérique septentrionale

*Clerus leucopsideus* [76: 8; pl. 1, fig. 6] Catalogne

*Clerus quadriguttatus* [76: 18; pl. 2, fig. 23] Caroline

*Clerus scabrosus* [76: 16; pl. 2, fig. 19] Afrique équinoxiale

*Clerus thoracicus* [76: 18; pl. 2, fig. 22] Caroline

*Clerus umbellatarum* [76: 5; pl. 1, fig. 2] Barbarie

*Bostrichus bidentatus* [77: 16; pl. 3, fig. 20] Syrie

*Bostrichus lineatus* [77: 18; pl. 3, fig. 23] Europe

*Bostrichus longicornis* [77: 15; pl. 3, fig. 18] Saint-Domingue

*Bostrichus rufipes* [77: 17; pl. 3, fig. 21] Paris

*Bostrichus rugosus* [77: 18; pl. 3, fig. 24] Amérique septentrionale

*Bostrichus trispinosus* [77: 16; pl. 3, fig. 19] Mésopotamie

*Scolytus destructor* [78: 5; pl. 1, fig. 4] Europe

*Scolytus frontalis* [78: 13; pl. 2, fig. 20] Amérique septentrionale

*Scolytus impressus* [78: 12; pl. 2, fig. 19] Paris

*Scolytus pusillus* [78: 14; pl. 2, fig. 23] Paris

*Scolytus quadridentatus* [78: 5; pl. 1, fig. 3] Amérique septentrionale

*Scolytus retusus* [78: 10; pl. 2, fig. 14] Paris

*Scolytus sexdentatus* [78: 11; pl. 2, fig. 15] Paris

*Scolytus spinosus* [78: 9; pl. 2, fig. 11] Java

*Scolytus terebrans* [78: 7; pl. 1, fig. 6] Amérique septentrionale

*Scolytus varius* [78: 11; pl. 2, fig. 17] France

*Bruchus biguttatus* [79: 20; pl. 3, fig. 27] France, îles de l’Archipel

*Bruchus bimaculatus* [79: 18; pl. 3, fig. 22] France

*Bruchus coryphae* [79: 16; pl. 2, fig. 18] Amérique septentrionale

*Bruchus fasciatus* [79: 20; pl. 3, fig. 25] environs de Paris

*Bruchus hibiscus* [79: 21; pl. 3, fig. 28] Amérique septentrionale

*Bruchus irroratus* [79: 21; pl. 3, fig. 29] Java

*Bruchus nebulosus* [79: 20; pl. 3, fig. 26] France

*Bruchus quinqueguttatus* [79: 15; pl. 2, fig. 16] Barbarie, aux îles de l’Archipel, sur les Cistes

*Bruchus tragacanthae* [79: 15; pl. 2, fig. 17] Perse

*Bruchus unicolor* [79: 17; pl. 2, fig. 20] Europe

*Bruchus varius* [79: 18; pl. 3, fig. 23] Europe

*Bruchus viciae* [79: 12; pl. 2, fig. 11] midi de la France

*Macrocephalus bidens* [80: 13; pl. 2, fig. 18] Saint-Domingue

*Macrocephalus bimaculatus* [80: 14; pl. 2, fig. 19] Géorgie

*Macrocephalus cinereus* [80: 4; pl. 1, fig. 2] Indes-Orientales

*Macrocephalus fasciatus* [80: 9; pl. 1, fig. 9] Amérique septentrionale, à la Géorgie

*Macrocephalus fuliginosus* [80: 11; pl. 2, fig. 13] Indes-Orientales

*Macrocephalus lugubris* [80: 13; pl. 2, fig. 17] Géorgie

*Macrocephalus maculatus* [80: 11; pl. 2, fig. 14] Indes-Orientales

*Macrocephalus marmoreus* [80: 12; pl. 2, fig. 16] Géorgie, en Caroline

*Macrocephalus murinus* [80: 12; pl. 2, fig. 15] Indes-Orientales

*Macrocephalus nebulosus* [80: 5; pl. 1, fig. 3] Cayenne

*Macrocephalus transversus* [80: 10; pl. 1, fig. 12] Indes-Orientales

*Macrocephalus tuberculatus* [80: 10; pl. 1, fig. 11] Afrique

*Macrocephalus variegatus* [80: 4; pl. 1, fig. 1] [Locality not indicated]

*Macrocephalus verrucosus* [80: 6; pl. 1, fig. 5] [Locality not indicated]
